# Memristive characteristic of an amorphous Ga-Sn-O thin-film device

**DOI:** 10.1038/s41598-019-39549-9

**Published:** 2019-02-26

**Authors:** Sumio Sugisaki, Tokiyoshi Matsuda, Mutsunori Uenuma, Toshihide Nabatame, Yasuhiko Nakashima, Takahito Imai, Yusaku Magari, Daichi Koretomo, Mamoru Furuta, Mutsumi Kimura

**Affiliations:** 1 Department of Electronics and Informatics, Ryukoku University, Seta, Otsu 520-2194 Japan; 2 Innovative Materials and Processing Research Center, High-Tech Research Center, Seta, Otsu 520-2194 Japan; 3 Graduate School of Materials Science, Nara Institute of Science and Technology, Takayama, Ikoma 630-0192 Japan; 4 MANA Foundry and MANA Advanced Device Materials Group, National Institute for Materials Science, Namiki, Tsukuba 305-0044 Japan; 5 Graduate School of Information Science, Nara Institute of Science and Technology, Takayama, Ikoma 630-0192 Japan; 6 Department of Material Chemistry, Ryukoku University, Seta, Otsu 520-2194 Japan; 7 Institute for Nanotechnology, Kochi University of Technology, Tosayamada, Kami 782-8502 Japan

## Abstract

We have found a memristive characteristic of an α-GTO thin-film device. The α-GTO thin-film layer is deposited using radio-frequency (RF) magnetron sputtering at room temperature and sandwiched between the Al top and bottom electrodes. It is found that the hysteresis loop of the flowing current (I) and applied voltage (V) characteristic becomes larger and stable after the one hundredth cycle. The electrical resistances for the high-resistance state (HRS) and low-resistance state (LRS) are clearly different and relatively stable. Based on various analysis, it is suggested that the memristive characteristic is due to the chemical reaction between the SnO_2_ and SnO blocked by AlO_x_ on the Al bottom electrode. It is marvelous that the memristive characteristic can be realized by such common materials, simple structures, and easy fabrication.

## Introduction

Amorphous metal-oxide semiconductor thin-film devices are widely utilized and also promising for various applications, because they can have excellent performances on individual demands by customization of materials, structures, fabrications, etc^1–4^. They can be fabricated at low temperature and produced on large area with low cost. Particularly, the research on α-GTO thin-film devices is focused for not only thin-film conductors^5–10^ but also thin-film transistors^11–13^ and other applications^14,15^. They do not include rare metals such as In, and industrial issues on resource depletion and supply anxiety can be solved. On the other hand, memristors are passive devices with electric resistance change relative to electric charge history^16^, and they are recently used for resistive random access memory (ReRAM)^17^, neural networks^18^, etc. However, the conventional memristors require expensive materials, structures, fabrications, etc.

In this study, we have found a memristive characteristic of an α-GTO thin-film device. We will show the device structure and fabrication process, introduce the memristive characteristic, analyze various properties, and discuss the working mechanism. It is marvelous that the memristive characteristic can be realized by such common materials, simple structures, and easy fabrication.

## Results

### α-GTO thin-film device

**Figure 1:**
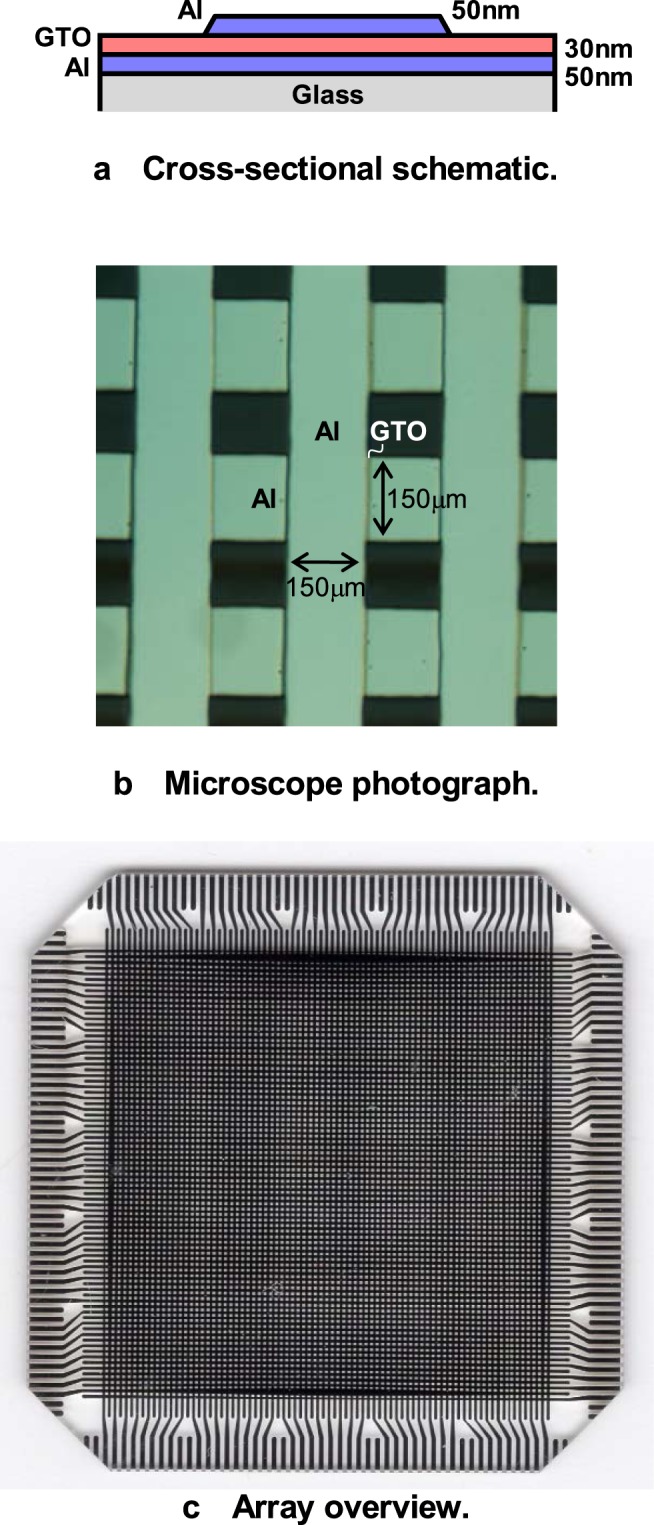
Device structure of the α-GTO thin-film device. (**a**) Cross-sectional schematic. (**b**) Microscope photograph. (**c**) Array overview.

The device structure of the α-GTO thin-film device is shown in Fig. 1. The α-GTO thin-film layer is deposited using radio-frequency (RF) magnetron sputtering at room temperature and sandwiched between the Al top and bottom electrodes. As a result, the α-GTO thin-film device is completed without additional annealing process, where the thickness of the GTO thin-film layer is 30 nm, the thicknesses of both the Al top and bottom electrodes are 50 nm, and the area of the α-GTO thin-film device is 150×150 μm corresponding to the cross point of the Al top and bottom electrodes.

### Memristive characteristic

**Figure 2:**
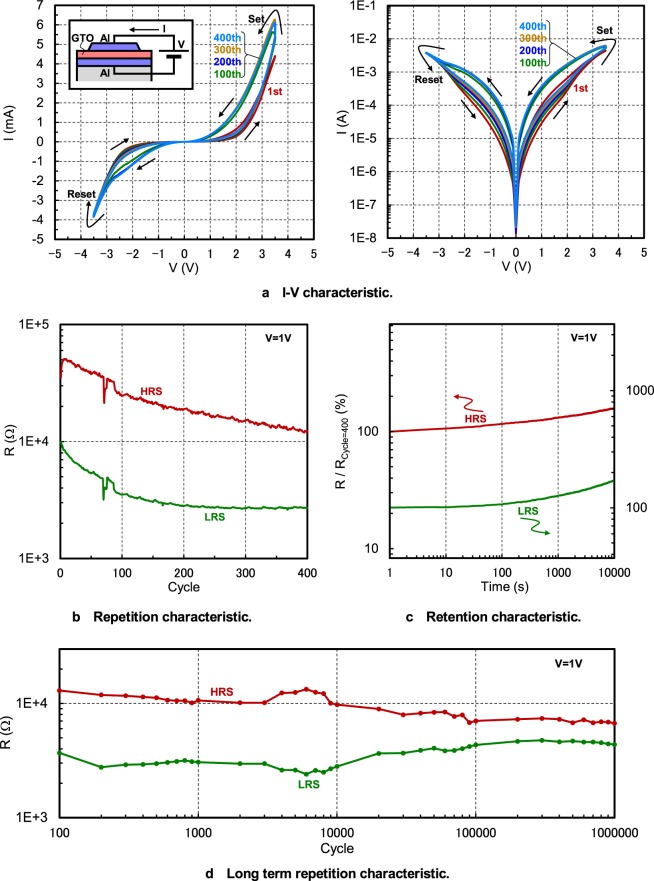
Memristive characteristic of the α-GTO thin-film device. (**a**) I-V characteristic. (**b**) Repetition characteristic. (**c**) Retention characteristic. (**d**) Long term repetition characteristic.

The memristive characteristic of the α-GTO thin-film device is shown in Fig. 2. Here, no forming process is done, because no clear forming behavior appears. The I-V characteristic is shown in Fig. 2a. It is found that although the hysteresis loop of the I-V characteristic is small for the first cycle, it becomes larger and stable after the one hundredth cycle. As V increases from 0 V to +3.5 V, I also increases. When V decreases from +3.5 V, I is larger than the previous I, which is called “set transition”. On the other hand, as |V| increase from 0 V to −3.5 V, |I| is also increases. When |V| decreases from −3.5 V, |I| is smaller than the previous |I|, which is called “reset transition”. It should be noted that although the absolute value of I is large because metal masks are used as written later and the device size is not so fine, it can be proportionally reduced when photolithography is used and the α-GTO thin-film device is downsized.

The repetition characteristic is shown in Fig. 2b. Here, the drops of electrical resistances at the 71th cycle seems due to some unclear accident. It is found that after the one hundredth cycle, the electrical resistances for the HRS and LRS are clearly different and relatively stable. The retention characteristic is shown in Fig. 2c. It is found that the electrical resistances for the HRS and LRS are relatively stable before 100 s, although they gradually increase after that, which should be solved in the future but is acceptable for some application as mentioned later. The long term repetition characteristic is shown in Fig. 2d. Until the ten thousandth cycle, the electrical resistances for the HRS and LRS are kept intact. Even after the one millionth cycle, the electrical resistances are surely different, although the resistance difference is gradually decreasing. In any case, we have found a memristive characteristic of an α-GTO thin-film device, which is reported for the first time, although memristive characteristics of amorphous In-Ga-Zn-O (α-IGZO) thin-film devices have been once reported^19–22^.

## Discussion

### Various properties

**Figure 3:**
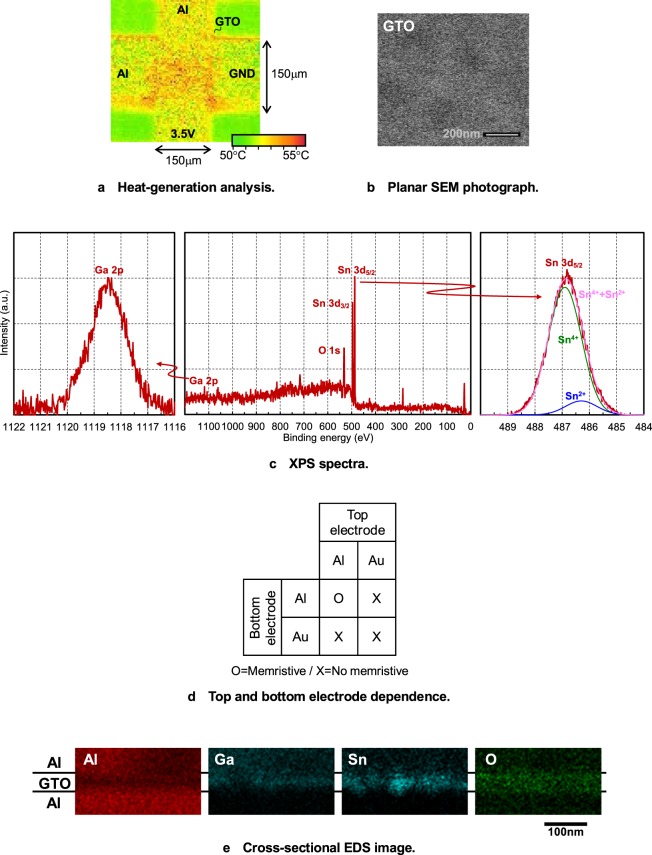
Various properties of the α-GTO thin-film device. (**a**) Heat-generation analysis. (**b**) Planar SEM photograph. (**c**) XPS spectra. (**d**) Top and bottom electrode dependence. (**e**) Cross-sectional EDS image.

Various properties of the α-GTO thin-film device are shown in Fig. 3. As shown in Fig. 3a, when V is +3.5 V, the temperature distribution are observed using infrared emission microscopy. It is found that the Joule heat is uniformly generated in the area of the α-GTO thin-film device, which means that there is no local filament. As shown in Fig. 3b, the surface shape is observed using planar scanning electron microscopy (SEM). It is found that the surface shape is very uniform, which means that there is no grain boundary and the α-GTO thin-film layer is truly in the amorphous phase, which is also confirmed using X-ray diffraction (XRD). Here, although the α-GTO thin-film layer is directly deposited on a quartz glass substrate, it is believed that the result is the same also even when it is sandwiched between the electrodes.

The X-ray photoelectron spectroscopy (XPS) spectra of the α-GTO thin-film layer is shown in Fig. 3c. It is found that the Ga 2p and Sn 3d peaks are observed, which means that the α-GTO thin-film layer is composed of GaO_x_ and SnO_x_, and the Sn 3d_5/2_ peak is the sum of the large Sn^4+^ peak and small Sn^2+^ peak, which means that SnO_2_ is more than SnO, although they are mixed, which is also similar to GTO thin films made under different conditions^13^. Because it was reported that SnO_2_ is n-type semiconductor and SnO is p-type semiconductor^23,24^, the α-GTO thin-film layer is overall n-type semiconductor, which is also confirmed by the facts that α-GTO thin-film transistors show n-type transistor characteristics^12^ and α-GTO thin-film thermoelectric devices show negative Seebeck coefficients^14^.

As shown in Fig. 3d, the top and bottom electrode dependence of the memristive characteristic is investigated, where Al and Au are used for the top and bottom electrodes. It is found that only when Al is used for both the top and bottom electrodes, the memristive characteristic appears, which means both the Al top and bottom electrodes play important roles. As shown in Fig. 3e, the elemental composition of the α-GTO thin-film device is investigated using energy dispersive X-ray spectrometry (EDS). Although it is investigated before the evaluation of the memristive characteristic, the result will be roughly the same even if it is done after the evaluation. It is found that Al is of course contained in the Al top and bottom electrodes, and Ga, Sn, and O are contained in the α-GTO thin-film layer, where the elemental composition is Ga:Sn:O = 10:25:65, and the ratio of Ga:Sn is roughly the same as that of the ceramic target written later. However, it should be noted that Ga and Sn are not diffused into the Al bottom electrode, but diffused into the top electrode, which suggest that AlO_x_ is made at the interface of the Al bottom electrode and block the diffusion of Ga and Sn, whereas it is not made at the interface of the Al top electrode. In addition, it should be also noted that O are contained even in the Al top and bottom electrodes.

Moreover, it is confirmed that the memristive characteristic of the α-IGZO thin-film device is not superior when the α-GTO thin-film layer is replaced by the α-IGZO thin-film layer and the otherwise same device structure and fabrication process are used, which suggest Sn plays an important role.

### Working mechanism

**Figure 4:**
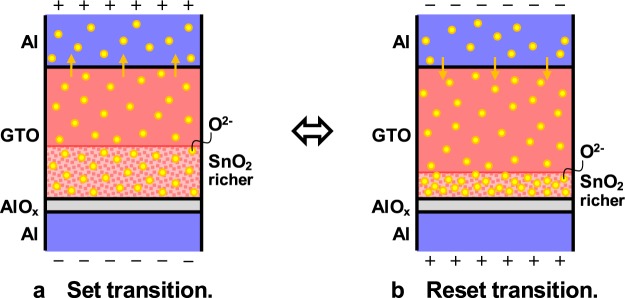
Working mechanism of the memristive characteristic. (**a**) Set transition. (**b**) Reset transition.

The working mechanism of the memristive characteristic is shown in Fig. 4. Based on the various analysis mentioned above, the following mechanism can be suggested. First, AlO_x_ is made at the interface of the Al bottom electrode during the beginning stage of the RF magnetron sputtering. Although the surface is exposed to the atmosphere, because the sputtering gas includes oxygen, the AlO_x_ is mainly made during the sputtering process. The AlO_x_ blocks the diffusion of any element, and the extra O^2−^ ions are concentrated in the lower part of the α-GTO thin-film layer, whereas AlO_x_ is not made at the interface of the Al top electrode.

The α-GTO thin-film layer is composed of GaO_x_ and SnO_x_, and SnO_2_ is more than SnO, although they are mixed, as aforementioned. Because SnO_2_ is n-type semiconductor and SnO is p-type semiconductor, the α-GTO thin-film layer is overall n-type semiconductor. When SnO_2_ replaces SnO, because the ratio of the n-type semiconductor increases and that of the p-type semiconductor decreases in the overall n-type semiconductor, the SnO_2_-richer layer is the high conductance layer. When +3.5 V is applied to the Al top electrode for the set transition, at the interface of the bottom electrode, the extra O^2−^ ions are drifted to the upper part of the α-GTO thin-film layer, where the O concentration increases and the SnO_2_-richer layer becomes thicker. Because the high conductance layer becomes thicker, the total conductance becomes higher. Incidentally, at the interface of the top electrode, it is believed that O^2−^ ions are drifted into the Al electrode, and there is neither concentration change of O^2−^ ions nor conductance change, which does not occur when Au is used for the top electrode because O^2−^ ions can hardly exist. On the other hand, when −3.5 V is applied for the reset transition, at the interface of the bottom electrode, the extra O^2−^ ions are returned to the lower part, where the SnO_2_-richer layer becomes thinner. Because the high conductance layer becomes thinner, the total conductance becomes lower. Incidentally, at the interface of the top electrode, O^2−^ ions are drifted back from the Al electrode into the α-GTO thin-film layer, and there is neither concentration change of O^2−^ ions nor conductance change again. The O in the Al top electrode originates from both that contained from the beginning and that drifted from the α-GTO thin-film layer, whereas the O in the bottom electrode originates only from that contained from the beginning.

It is believed that the memristive characteristic is due to the chemical reaction between the SnO_2_ and SnO blocked by AlO_x_ on the Al bottom electrode, which is quite different from that of an α-IGZO thin-film device^19–22^. This working mechanism is what is suggested by a kind of elimination method from the various analysis mentioned above, and we will continue to analyze further in the future.

## Conclusion

We have found a memristive characteristic of an α-GTO thin-film device. Al bottom electrodes were deposited, an α-GTO thin-film layer was deposited using RF magnetron sputtering at room temperature, Al top electrodes were deposited, and the α-GTO thin-film device was completed, where the αα-GTO thin-film layer was sandwiched between the Al top and bottom electrodes. V was scanned many times, and I was measured. It was found that the hysteresis loop of the I and V characteristic became larger and stable after the one hundredth cycle. The electrical resistances for the HRS and LRS were clearly different and relatively stable. Based on various analysis, it was suggested that the memristive characteristic was due to the chemical reaction between the SnO_2_ and SnO blocked by AlOx on the Al bottom electrode. It is marvelous that the memristive characteristic can be realized by such common materials, simple structures, and easy fabrication. Although the hysteresis loop is not so huge, it is rather convenient for some applications such as analog memories and neural networks. Particularly, for the neuromorphic application, not so high speed operation and surpassing endurance are required, and on the contrary, insufficient endurance is preferable for some cases^19,22^. Therefore, the memristive characteristic obtained in this study is acceptable. Instead, because astronomical number of processing elements must be integrated, it is useful that the memristive characteristic can be realized by such common materials, simple structures, and easy fabrication. As for the ReRAM application, the long term repetition characteristic is not sufficient and should be improved in the future.

## Methods

### α-GTO thin-film device

First, a quartz glass substrate is used, and Al bottom electrodes are deposited using vacuum evaporation through a metal mask to form horizontal bus lines, whose thickness is 50 nm and width is 150 μm. Next, an α-GTO thin-film layer is deposited using RF magnetron sputtering with a ceramic target of Ga:Sn = 1:3 and diameter of 2 inch, sputtering gas of Ar:O_2_ = 20:1 sccm, plasma power of 60 W, and substrate temperature of room temperature, whose thickness is 30 nm. Finally, Al top electrodes are deposited to form vertical bus lines, whose thickness is 50 nm and width is 150 μm.

### Memristive characteristic

#### I-V characteristic

V is applied between the Al top and bottom electrodes and scanned from −3.5 V to +3.5 V and vice versa many times, and I flows through the α-GTO thin-film device and is measured.

#### Repetition characteristic

The HRS is defined as the state after the reset transition, whereas the LRS is defined as the state after the set transition, and the electrical resistances when V is +1 V for the HRS and LRS are plotted.

#### Retention characteristic

V of +1 V is continuously applied, and the electrical resistances are measured for the HRS and LRS.

#### Long term repetition characteristic

The electrical resistances for the HRS and LRS are recorded for the 100th, 200th, ···, 1,000th, ···, 1,000,000th cycle, which are indicated using closed circles in the graph plots in Fig. 2d. V is only applied for the other cycles, and the electrical resistances are not recorded.
